# Face individual identity recognition: a potential endophenotype in autism

**DOI:** 10.1186/s13229-020-00371-0

**Published:** 2020-10-21

**Authors:** Ilaria Minio-Paluello, Giuseppina Porciello, Alvaro Pascual-Leone, Simon Baron-Cohen

**Affiliations:** 1 Department of Psychology, Sapienza University of Rome, Rome, Italy; 2 IRCCS Fondazione Santa Lucia, Rome, Italy; 3 Institute of Cognitive Sciences and Technologies, National Research Council, Rome, Italy; 4 Hinda and Arthur Marcus Institute for Aging Research and Center for Memory Health, Hebrew SeniorLife, Boston, MA USA; 5 Department of Neurology, Harvard Medical School, Boston, MA USA; 6 Guttmann Brain Health Institute, Institut Guttmann de Neurorehabilitació, Universitat Autonoma de Barcelona, Badalona, Spain; 7 Autism Research Centre, Department of Psychiatry, University of Cambridge, Cambridge, UK

**Keywords:** Autism, Individual identity recognition, Face memory, Prosopagnosia, Endophenotype, Heterogeneity, Social memory, Theory of mind, Emotion recognition

## Abstract

**Background:**

Face individual identity recognition skill is heritable and independent of intellectual ability. Difficulties in face individual identity recognition are present in autistic individuals and their family members and are possibly linked to oxytocin polymorphisms in families with an autistic child. While it is reported that developmental prosopagnosia (i.e., impaired face identity recognition) occurs in 2–3% of the general population, no prosopagnosia prevalence estimate is available for autism. Furthermore, an autism within-group approach has not been reported towards characterizing impaired face memory and to investigate its possible links to social and communication difficulties.

**Methods:**

The present study estimated the prevalence of prosopagnosia in 80 autistic adults with no intellectual disability, investigated its cognitive characteristics and links to autism symptoms’ severity, personality traits, and mental state understanding from the eye region by using standardized tests and questionnaires.

**Results:**

More than one third of autistic participants showed prosopagnosia. Their face memory skill was not associated with their symptom’s severity, empathy, alexithymia, or general intelligence. Face identity recognition was instead linked to mental state recognition from the eye region only in autistic individuals who had prosopagnosia, and this relationship did not depend on participants’ basic face perception skills. Importantly, we found that autistic participants were not aware of their face memory skills.

**Limitations:**

We did not test an epidemiological sample, and additional work is necessary to establish whether these results generalize to the entire autism spectrum.

**Conclusions:**

Impaired face individual identity recognition meets the criteria to be a potential endophenotype in autism. In the future, testing for face memory could be used to stratify autistic individuals into genetically meaningful subgroups and be translatable to autism animal models.

## Background

Autism is a highly hereditable, lifelong, neurodevelopmental condition characterized by difficulties in social communication and interaction, alongside unusually restricted and repetitive behavior and interests, sensory hypersensitivity, and difficulties adjusting to unexpected change [1]. Autism occurs in at least 1% of the population [2] and is associated with high levels of poor mental health which could be reduced by a better and earlier intervention [3]. Clinical, etiological, and genetic heterogeneity in autism poses challenges to the discovery of causes and the development of effective interventions for autism [4–6]. Heterogeneity could be addressed via an endophenotype-based stratification approach which would accelerate the identification of genetic underpinnings and specific interventions [7, 8]. An endophenotype approach in autism is complicated by the fact that phenotypic expression in autism changes developmentally over time, depends on symptom severity, and varies in the presence of intellectual disability (ID) which commonly co-occurs with autism [9, 10]. Researchers have proposed interesting behavioral and neural endophenotypes in autism [8, 11], including language delay [12], gazing at social scenes [13], scores on the Social Responsiveness Scale [14], and white matter structure [15]. Many other proposed endophenotypes in autism do not meet the definition of endophenotype they adopt [16] as, for example, they lack evidence of heritability [17–20]. Weaknesses of many endophenotypes proposed so far include the unknown prevalence in autism or relevance to a small subgroup of autistic individuals [15, 21, 22], reduced proximity to gene action (i.e., being under genetic influences of unknown or small effect sizes that are comparable to those of autism itself) [23], unknown or high genetic complexity [14, 24], lack of known or envisaged neurobiological bases [13], and limited translatability to animal models [12, 14, 23]. Such weaknesses are not specific to autism endophenotype literature but apply to endophenotypes of psychiatric conditions in general [25–27]. Individual identity recognition (IIR) could be a potential new autism endophenotype devoid of most of the weaknesses described above.

The ability to recognize another individual is crucial for social interaction [28], emerges very early in development [29, 30], is conserved across species [28], and is linked to the oxytocin (OXY) system [31, 32]. Humans, like other primates, recognize other individuals mostly by their face [33–35], which has evolved to signal individual identity [36]. Identity recognition is challenged by a face’s intrinsic (e.g., age, facial expressions/movements) and extrinsic (e.g., visual perspective, luminosity) identity-invariant changes and might benefit from plastic face representations [37–39]. Humans show large individual differences in their face IIR ability [40], including 2–3% of individuals in the general population who report severe difficulties recognizing identity from faces in everyday life [41, 42]. This face-blindness condition, known as developmental prosopagnosia (DP), is not associated with brain damage or deficits in low-level vision, can run in families, is likely polygenic [43], and the OXY system seems to play a role in it [44, 45]. Although there are no formal diagnostic criteria for DP, it is generally agreed diagnostic assessment should primarily involve objective measures of face IIR. The Cambridge Face Memory Test (CFMT) [46] is considered the gold standard memory test for unfamiliar faces [47–49] with a clinical cut-off score for prosopagnosia (see the “Methods” section).

While face perception in general, ranging from face detection to emotion recognition, has been extensively studied in autism [50, 51], face memory/IIR has been the focus of few well-controlled studies. In fact, studies that attempted to investigated face memory/IIR in autism often used tasks that suffered from (1) familiarity confounds (i.e., the task could be solved by recognizing whether an individual is seen before or novel) [52–55], (2) no (or very short) retention time of the memory trace related to facial identity [56–58], (3) face stimuli that include non-facial features (e.g., hair, clothes) [56, 59, 60] which allow correct performance even when facial internal features are covered [61], and (4) matching tasks with identical target and test face images, that therefore could be solved based on feature matching strategies [54, 62].

Overall, recent reviews of the available evidence have shown that, whenever memory is involved, autistic individuals as a group have reduced face identity recognition skills compared to matched neurotypical controls [51, 63] and that their face memory difficulties seem both process (e.g., memory vs. perception) and social-domain (e.g., faces and bodies vs. houses) specific [64]. To the best of our knowledge, no study has reported the prevalence of clinical impairment in face memory (i.e., prosopagnosia) in autism. In fact, those studies that used the CFMT, in many cases did not report group means and standard deviations but just case-control statistics [65–69]. When they did report group means, however, they did not report the percentage of participants meeting the CFMT prosopagnosia cut-off [70–74]. An exception is the study by Hedley and colleagues [75] who reported that 8 out of their 34 AUT adults (i.e., 24%) were prosopagnosic according to the CFMT.

No study has tested face memory in a larger group of autistic adults, that is, at an age when difficulties in face memory, if present, are most evident [70]. Further, no previous study has considered face memory difficulties in terms of autism within-group variability rather than mean case-control differences, addressing the heterogeneity in performance found in autism.

Face IIR has the potential to be an autism endophenotype because it is highly heritable [76–78], independent of intellectual ability [78, 79], and difficulties in IIR are more common in family members of autistic individuals [59, 80, 81]. Future autism research could benefit from the fact that IIR is measurable early in development [30, 82] and in individuals with ID [83], may be linked to OXY polymorphisms in families with an autistic child [60] (for different findings in neurotypical participants, see [84]), and is translatable to mice [85, 86].

Here, we estimated the prevalence of prosopagnosia in autism, characterized it with respect to related face perceptual processes, and investigated its links to autism symptom severity, personality traits, and difficulties in mental state understanding from the eye region.

## Methods

### Participants

We tested 80 autistic (AUT) adults (16 females, mean age = 31.3 ± 11.9 years, age range = 18–73 years) with no ID (IQ- percentile = 78 ± 29, range = 4–100, *N* = 74 of which Raven’s SPM mean percentile = 91 ± 15, range = 38–100, *N* = 31; Wechsler scales mean full-scale IQ percentile = 69 ± 32, range = 4–100, *N* = 43), and 80 neurotypical controls (NT) (16 females, mean age = 28.8 ± 9.1 years, age range = 18–56 years) with no ID (IQ percentile = 92 ± 13, range = 21–100, *N* = 69, of which Raven’s SPM mean percentile = 93 ± 14, range = 21–100, *N* = 47; Wechsler scales mean full-scale IQ percentile = 89 ± 12, range = 50–100, *N* = 22) matched for age (*t* test with separate variance estimates, *t*(147.7) = 1.47, *p* = 0.14, 95% CI [− 5.75, 0.85]; Levene’s test *F*(1,158) = 4.43, *p* = 0.04), sex, and country of residency (UK, USA, Italy). Although we did not have IQ measures for 6 AUT and 11 NT participants, given that they all completed high school and in some cases were enrolled in college-level education, we could assume they too did not have ID.

Autism diagnosis was made by a professional expert in autism according to DSM-IV criteria and confirmed via the Autism Diagnostic Observation Schedule (ADOS) [87] and the Autism Diagnostic Interview Revised (ADI-R) [88] by a certified clinician (Table 1). Intellectual ability was assessed via either the Wechsler Abbreviated Scale of Intelligence [89], the Wechsler Adult Scale of Intelligence IV [90], or the Raven’s Standard Progressive Matrices [91] (Table 1). NT participants did not have a neurological or psychiatric condition, autistic first-degree relatives, or an autism spectrum quotient questionnaire score above the autism cut-off [92]. Participants were recruited because they took part in other experimental studies [93–95]; therefore, they were not preselected based on their interest or skills in identity recognition. The study was approved by local Institutional Review Boards, adhered to the declaration of Helsinki, and all participants gave informed consent before participation.

**Table 1:** Characteristics of the autistic sample

	ADOS CSS	ADOS SA + RRB	ADOS Comm + SocInt	ADI Comm	ADI Soc Int	ADI RRB	ADI Abn Dev	IQ percentile	AQ
Cut-off		8	7	8	10	3	3		31
**AUT**	7 ± 3 *N* = 67	13 ± 5 *N* = 67	10 ± 4 *N* = 69	16 ± 5 *N* = 55	19 ± 6 *N* = 55	6 ± 3 *N* = 55	2 ± 1 *N* = 55	78 ± 29 *N* = 74	31 ± 9 *N* = 78

*ADOS* Autism Diagnostic Observation Schedule, *CSS* Calibrated Severity Score, *SA* Social Affective, *RRB* Restricted Repetitive Behavior, *Comm* Communication, *Soc Int* Social Interaction, *ADI* Autism Diagnostic Interview, *Abn Dev* abnormal development, *IQ* intelligence quotient, *AQ* autism quotient

### Procedure

Participants performed computer-based versions of a standardized battery of tests and questionnaires either onsite or online (6% of NT and 14% of AUT participants completed between one and three questionnaires/tests online). All participants completed the Cambridge Face Memory Test (CFMT) [46] upright, and almost all autistic participants completed the other tests and questionnaires (sample size is reported for each test).

### Tests

#### *The Cambridge Face Memory Test (CFMT)* [46] *upright and inverted*

The CFMT is a computer-based test that uses a three-alternative forced-choice paradigm with the unlimited response time. Participants have to memorize and recognize 6 individuals. The test is divided into 3 parts of increasing difficulty. In the first part (18 trials), participants view each individual from three different angles and have to recognize him in three consecutive trials (correct images are identical to the studied ones). In the second part (30 trials), the to-be-recognized individual can be any of the 6 previously learned ones, now presented with different angles and/or lighting. The third part (24 trials) has the same structure as the second one but Gaussian visual noise is added to the images in order to make participants rely more on holistic (vs. feature-based) face processing [96]. A number of correct answers at or below 42 (out of 72), which corresponds to 2 standard deviations from the mean, are indicative of prosopagnosia, while chance level corresponds to 24 correct responses [46, 47]. The CFMT has well-controlled stimuli (e.g., including only facial features, the same individual is presented with identity invariant changes), is unidimensional, highly reliable, has high discriminant and convergent validity, is precise over a wide range of ability levels [47, 97], and can be reliably administered online [98, 99]. Performance on the CFMT is heritable [100] and has little or no correlation with general intelligence [78, 79]. The AUT group also took the CFMT with face stimuli presented upside down [47, 101]. AUT participants took the upright and the inverted CFMT tests in counterbalanced order, one at the beginning and one at the end of the experiment, to reduce carry-over effects. Better performance for upright vs. inverted faces is known as the face inversion effect, which is interpreted in favor of typical holistic (vs. feature-based) processing of faces [102]. In order to account for age-dependent decline in performance, we used Bowles and colleagues [47] second-order polynomial fit in conjunction with the standard deviations of the residuals of that fit to calculate age-standardized prosopagnosia cut-off scores for participants over 49 years of age.

#### *The Cambridge Face Perception Test (CFPT)* [103]

The CFPT is a computer-based test where each of its 16 trials involves limited time (i.e., 40 s in the version used here) to sort 6 front-facing faces according to their similarity to a target face presented with a ¾ profile. Each of the 6 faces was created by morphing a different individual with the target face by varying degrees (28%, 40%, 52%, 64%, 76%, and 88%). In half of the trials, faces are presented upright, while in the other half of the trials, faces are presented inverted. For each trial, the order in which the participant arranged the morphed faces is scored by summing the deviations of each morphed face from its correct position. The CFPT error score corresponds to the sum of scores of upright trials. The higher the error score, the worse the participants’ performance. Perfect performance corresponds to an error score of 0, while chance performance corresponds to an error score of 93.3. The CFPT is considered a measure of face perception skills with no memory demand, as the target and the morphed faces are visible during the task and, therefore, do not need to be memorized. Not all individuals with DP perform worse than controls on the CFPT [47, 104, 105], just those with apperceptive prosopagnosia.

#### *The Reading the Mind in the Eyes Test (RMET)* [106]

The RMET is a test where participants have to recognize mental states (including complex emotions) from 36 photographs of the eye region of individuals varying in sex and age. The RMET has good reliability [107], autistic individuals consistently perform less well than matched neurotypical controls [108–112] and do not show the neurotypical advantage of female vs. male participants [108]. A large online study of the RMET in over 80.000 individuals confirmed the neurotypical sex difference (female advantage) and identified a single nucleotide polymorphism associated with performance [113]. Individuals with DP perform similarly to controls on the RMET [103, 114–116].

### Questionnaires

#### *The Autism Spectrum Quotient (AQ)* [92]

The AQ is a 50-item self-report questionnaire measuring the number of autistic traits across five domains: communication, social skills, attention switching, imagination, and attention to detail. The respondent rates how strongly they agree or disagree with each statement, using a four-point scale. Total score ranges from 0 to 50 and scores above 31 are indicative of autism. Scores between 23 and 28 are considered Broad Autism Phenotype (BAP), between 29 and 34 Medium Autism Phenotype (MAP), and above 34 Narrow Autism Phenotype (NAP) [117]. A large online study of the AQ confirmed the sex difference (typical males score higher on average than typical females) and the STEM effect (those working in Science, Technology, Engineering, and Math score higher on average than those who do not) in half a million people [118]. A recent, even larger online study of over 600,000 people from the general population and 36,000 autistic people, confirmed the case-control difference, the sex difference, and the STEM effect [119], using a short form of the AQ.

#### *The twenty-item Prosopagnosia Index (PI20)* [120]

The PI20 is a 20-items self-report questionnaire proposed by its authors as a measure of prosopagnosic traits [121]. Respondents indicate on a five-point Likert scale how much they agree with statements describing their face identity recognition abilities and experience in everyday life. Total score ranges from 20 to 100 and a score over 64 is considered indicative of prosopagnosia [120]. The PI20 score correlates with performance on the CFMT in the general population and in DP [120–122] and distinguishes DPs from controls [120, 123, 124]. The need for DP diagnostic assessment to include a self-report questionnaire assessing awareness of everyday difficulties in face memory is debated [48, 49, 125].

#### *The Interpersonal Reactivity Index (IRI)* [126]

The IRI is a 28-item self-report questionnaire with four subscales each said to be measuring an independent empathy component. Subscales include *perspective taking*, which measures the ability to adopt another person’s view point; *empathic concern*, which measures the tendency to respond with warm, compassionate feelings for others; *fantasy*, which measures the tendency to identify with fictional characters; and *personal distress*, which measures a self-oriented negative arousal/discomfort response to another person’s distress/negative experience. Participants indicate on a five-point Likert scale how much they agree with each statement.

#### *The Toronto Alexithymia Scale (TAS)* [127]

The TAS is a 20-item self-report questionnaire with three subscales each tapping a component of alexithymia: difficulty identifying feelings, difficulty describing feelings, and externally oriented thinking. Total scores range between 20 and 100, with higher scores indicating more alexithymic traits. Sixty-one is the cut-off score for high alexithymia [128]. The TAS has good internal consistency and good test-retest reliability [127]. Alexithymia seems highly prevalent in people on the autistic spectrum compared to the general population, and it seems to play a role in autism emotion recognition skills and empathic response [129].

### Statistical analysis

Before running each statistical test, we checked whether its assumptions were met. Before running Student’s *t* tests, we checked for homogeneity of variances via Levene’s test and when significant we considered separate variances estimates. Before running moderation analysis, we checked for multicollinearity (via variance inflation factor and tolerance), independence of residuals (via Durbin–Watson statistic), linearity and homoscedasticity (via visual inspection of the standardized residual vs. standardized values scatter plot), and homogeneity of variances (via Levene’s test). We considered a dependent variable to be normally distributed if Shapiro-Wilks test was not significant (*p* > 0.05) or if its absolute SME-standardized *Z*-Skewness and *Z*-Kurtosis were considered normal (i.e., *Z*-Skewness and *Z*-Kurtosis < 1.96 at *p* < 0.05) [130]. In case of normal distribution, we reported parametric tests; otherwise, non-parametric tests were used. Specifically, when data distribution was not normal and/or sample sizes were different, we used robust statistics [131]. For each statistical test, we reported relevant statistical indices including the distribution’s parameters and their degrees of freedom (df), the sample size used (N), mean or median, standard deviation (SD), probability (p), confidence intervals (CI), and effect size. We did not randomize the recruitment selection of autistic (nor matched NT) participants as recruitment of autistic participants was not easy, and our main goal was to have the largest possible sample size.

We did not find outliers in correlation and regression analyses as no participant met at least two between Mahalanobis distance, Cook’s distance and Leverage cut-offs [130]. Statistical analysis was run via SPSS (IBM) and PROCESS plugin [132], STATISTICA (StatSoft, Inc. 2007), and R (R Development Core Team 2013, packages *WRS2, RVAideMemoire, ggplot2*).

## Results

### Intellectual ability and autistic traits in autistic and neurotypical participants

Comparison of available autistic (74 out of 80) and neurotypical (69 out of 80) IQ percentile scores via robust Yuen’s test (WRS2, R-package) with default trimmed value of 0.2 provided Ty(54.53) = 2.42, *p* = 0.02, trimmed mean difference = 7.74, 95% CI [1.33, 14.15], and explanatory measure of effect size = 0.45, 95% CI [0.18, 0.71]. Therefore, based on the available data, NT (92 ± 13) had higher IQ percentiles than AUT (78 ± 29) participants.

Results of the AQ showed that 41% of AUT participants scored within the narrow autism phenotype range (vs. 0% of NT), 27% within the medium autism phenotype range (vs. 1% of NT), 13% within the broad autism phenotype range (vs. 8% of NT) and 19% outside the broad autism phenotype (vs. 91% of NT) [117]. As expected, AUT (31.01 ± 9.02, *N* = 78) had higher AQ scores than NT (14.86 ± 6.01, *N* = 78) participants, *t* test with separate variance estimate *t* (134) = 13.17, *p* = 6.20e^-26^, 95% CI [13.73, 18.58]; Levene’s test *F*(1,154) = 9.21, *p* = 0.003.

### Prosopagnosia is more common in autism than in controls

**Fig. 1:**
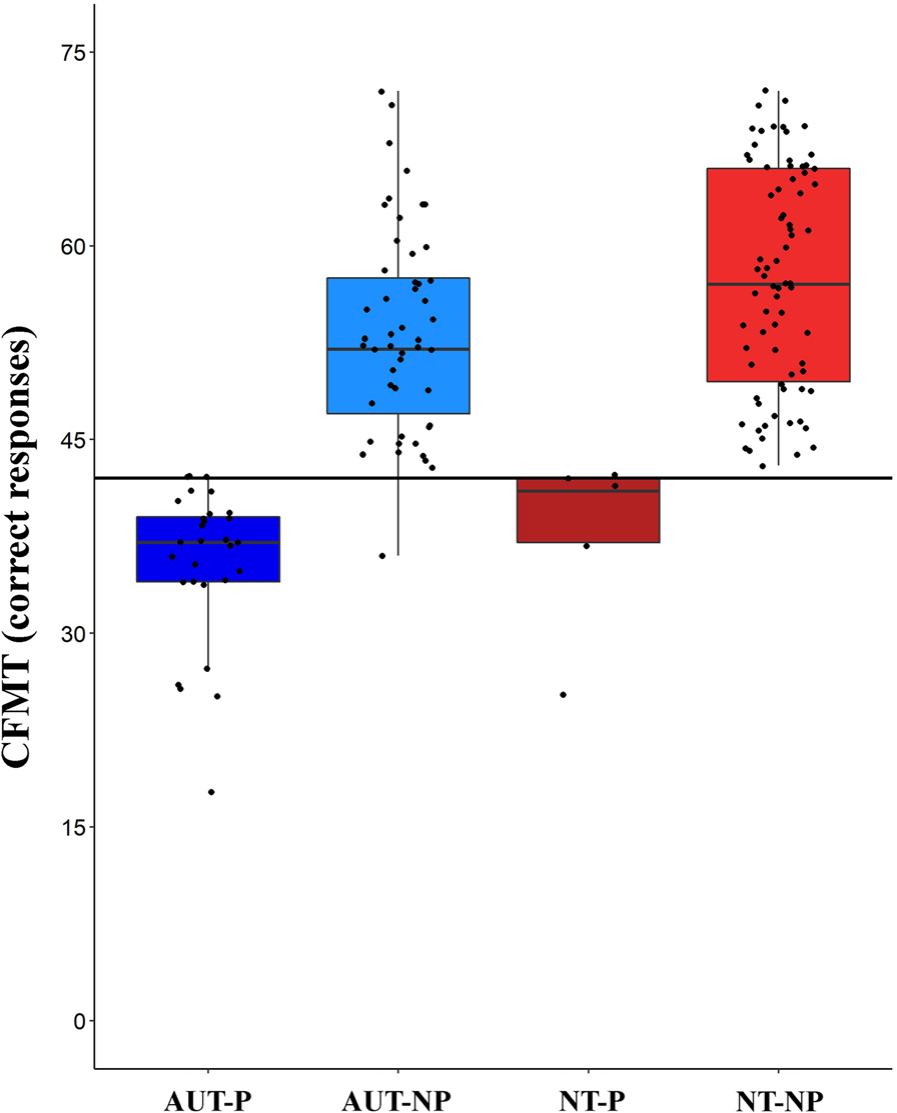
The prevalence of prosopagnosia in autism. Performance (number of correct responses) at the Cambridge Face Memory Test (CFMT) for 160 participants plotted as a function of their diagnostic group (autistic-AUT vs. neurotypical-NT) and prosopagnosia (prosopagnosic-P vs. non-prosopagnosic-NP). Thicker horizontal lines represent the medians, boxes the interquartile ranges, and whiskers the maximum and minimum values. The solid horizontal black line is the CFMT clinical cut-off for prosopagnosia (i.e., 42). AUT-P: dark blue, AUT-NP: light blue, NT-P: dark red, NT-NP: light red boxes. Black dots represent individual data points

We found that prosopagnosia was more common in autism: 36% of AUT met the clinical cut-off for prosopagnosia, while this was the case for only 6% of NT. Autism diagnosis was significantly associated with prosopagnosia *χ*^2^(1) = 21.51, *p* < 0.001, 95% CI [0.18, 0.42], that is, based on the odds ratio, the odds of being prosopagnosic were 8.5 times higher for AUT individuals than for NT (Fig. 1). Please see Table 2 for groups’ performance scores on the CFMT and note from Fig. 1 that all groups showed some degree of variation in the number of correct responses on the CFMT. As evident in every figure, although one participant scored below the CFMT cut-off (i.e., 42), they were assigned to the AUT-NP group as they did not meet their age-standardized prosopagnosia cut-off.

**Table 2:** Group performance at the Cambridge Face Memory Test (CFMT) of prosopagnosic (P) and non-prosopagnosic (NP) autistic (AUT) and neurotypical (NT) participants

CFMT (prosopagnosia cut-off = 42)	*N*	mean number of correct responses (over a total of 72)	SD	Range
**NT** (NT-NP + NT-P)	80	56.20 95% CI [54.03, 58.37]	9.76 95% CI [8.45, 11.56]	25–72
**NT-NP**	75	57.45 95% CI [55.48, 59.42]	8.57 95% CI [7.38, 10.21]	43–72
**NT-P**	5	37.40 95% CI [28.42, 46.38]	7.23 95% CI [4.33, 20.78]	25–42
**AUT** (AUT-NP + AUT-P)	80	46.85 95% CI [44.36, 49.34]	11.20 95% CI [9.69, 13.26]	18–72
AUT-NP*	51	53.64 95% CI [51.49, 55.79]	7.55 95% CI [6.31, 9.41]	43–72
**AUT-P**	29	35.52 95% CI [33.28, 37.75]	5.87 95% CI [4.66, 7.94]	18–42

*NT* neurotypical participants, *AUT* autistic participants, *P* prosopagnosic, *NP* non-prosopagnosic, *CFMT* Cambridge Face Memory Test, *SD* standard deviation, *CI* confidence interval

*AUT-NP mean, SD, and range do not include a participant who scored 36 and was assigned to the AUT-NP group as they did not meet their age-standardized prosopagnosia cut-off

### Clinical and personality trait measures do not distinguish between autistic individuals with and without prosopagnosia

Autistic prosopagnosic (AUT-P) and autistic non-prosopagnosic (AUT-NP) individuals did not differ in their diagnostic symptom severity, assessed via the ADOS Calibrated Severity Score (CSS) [133] and the ADI-R total score (i.e., Communication, Social Interaction, Restricted Repetitive Behaviors, and Developmental Abnormalities sum of scores), number of autistic traits assessed via the AQ questionnaire [92], level of general intelligence assessed via Raven’s progressive matrices or Wechsler adult intelligence scales, mental state recognition from the eye region assessed via the RMET [106], perspective taking (PT) and empathic concern (EC) assessed via the IRI [126], alexithymia assessed via the TAS [127], and self-report prosopagnosia assessed via the PI20 questionnaire [120] (see Table 3).

**Table 3:** Comparisons between autistic participants with (AUT-P) and without (AUT-NP) prosopagnosia

	Group	*N*	Mean	SD	SE	t (df)/T_**y**_(df)	p	95% CI	Cohen’s d/Yuen’s effect size
**ADOS CSS**	AUT-NP	43	6.40	2.55	0.39				
	AUT-P	24	7.08	2.60	0.53	− 1.05(65)	0.30	− 1.99,0.62	− 0.27
**ADI TOT**	AUT-NP	38	43.16	11.28	1.83				
	AUT-P	17	45.77	12.95	3.14	− 0.76 (53)	0.45	− 9.52, 4.31	− 0.22
**AQ**	AUT-NP	51	30.86	9.53	1.33				
	AUT-P	27	31.30	8.12	1.56	0.13 (39)	0.89	− 3.54, 4.04	0.07
**IQ**	AUT-NP	49	80.56	26.86	3.84				
	AUT-P	25	73.68	31.73	6.35	0.87 (17.94)	0.35	− 8.52, 23.09	0.21
**RMET**	AUT-NP	50	0.65	0.16	0.02				
	AUT-P	26	0.60	0.19	0.04	0.95 (22.3)	0.35	− 0.07, 0.17	0.18
**PI20**	AUT-NP	42	55.00	15.51	2.39				
	AUT-P	21	56.57	16.07	3.51	0.40 (23.59)	0.69	− 12.11, 819	0.1
**PT**	AUT-NP	50	13.22	6.07	0.86				
	AUT-P	26	12.46	5.57	1.09	0.69 (74)	0.49	− 1.84, 3.79	0.17
**EC**	AUT-NP	50	16.08	5.30	0.75				
	AUT-P	26	16.27	6.27	1.23	− 0.004 (74)	1.00	− 2.70, 2.69	− 9.37e−4
**TAS**	AUT-NP	44	50.32	10.60	1.60				
	AUT-P	23	59.09	10.99	2.29	1.85 (65)	0.07	− 0.41, 10.57	− 0.44

Cohen’s *d* effect size is interpreted as 0.2 (small), 0.5 (medium), 0.8 (large); (_*_) Yuen’s effect size is interpreted as 0.10 (small), 0.30 (medium), and 0.50 (large)

*t* = Student t, (_*_) T_*y*_ *=* Yuen’s *T*, *SD* standard deviation, *SE* standard error, *df* degrees of freedom, *CI* confidence interval, *AUT-P* autistic prosopagnosic, *AUT-NP* autistic non-prosopagnosic, *ADOS* Autism Diagnostic Observation Schedule, *ADI TOT* Autism Diagnostic Interview Total score (i.e., Communication + Social Interaction + Restricted Repetitive Behaviour + Developmental Abnormalities), AQ^*^ autism quotient, IQ^*^ Intelligence Quotient, RMET^*^ Reading the Mind in the Eyes Test, *PI20* Twenty-item Prosopagnosia Index, *PT* Perspective Taking, *EC* Empathic Concern, *TAS* Toronto Alexithymia Scale

### Face individual identity recognition is linked to mental state recognition only in autistic individuals with prosopagnosia

**Fig. 2:**
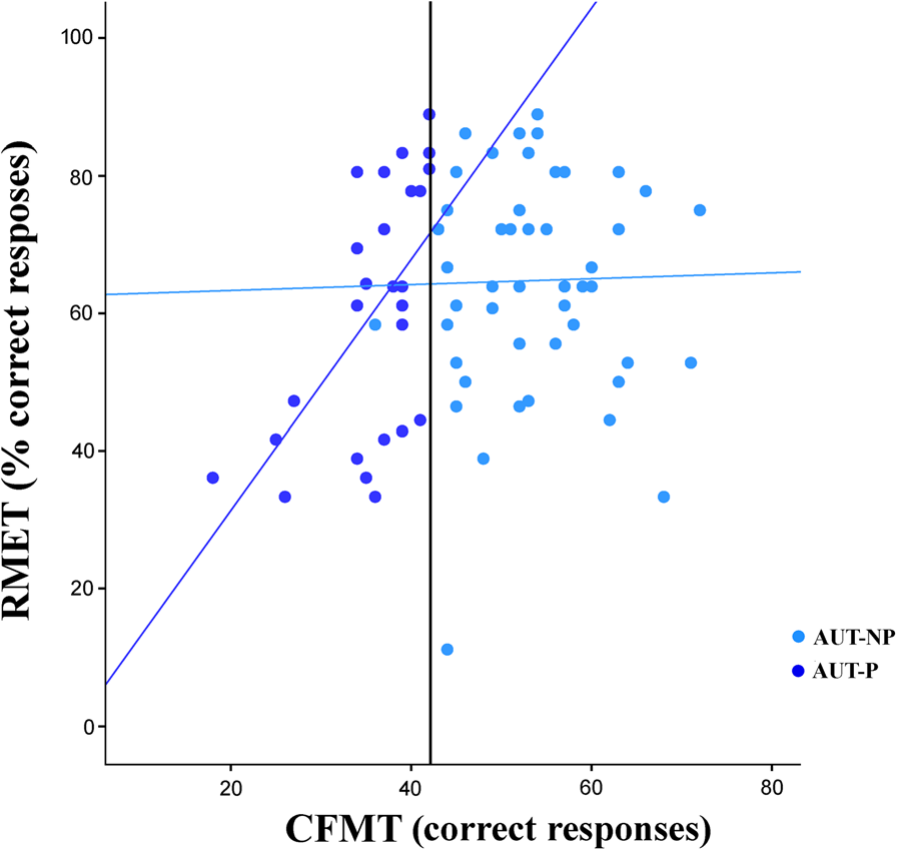
Prosopagnosia moderates the influence of identity recognition on mental state understanding. In autistic participants who are prosopagnosic (AUT-P, dark blue circles), face memory skill at the CFMT predicted their ability to understand another person’s mental states at the RMET, while this was not the case for autistic participants who are not prosopagnosic (AUT-NP, light blue circles). The dark blue solid line represents a significant regression line for the AUT-P group, while the light blue line represents nonsignificant regression line for the AUT-NP group. The black solid line represents CFMT cut-off score (i.e., 42)

We completed a moderation analysis to investigate the role of face IIR over mental state recognition in the two groups of prosopagnosic and non-prosopagnosic AUT participants. Moderation analysis, *R*^2^ = 0.16, *F* (3, 72) = 4.48, *p* = 0.006, revealed that the interaction between identity recognition, assessed via the CFMT, and whether AUT participants were prosopagnosic or non prosopagnosic, predicted participants’ ability to infer another person's mental states by looking at their eye region, assessed via the RMET, *b* = 0.02, SE = 0.006, *t* = 3.00, *p* = 0.004, 95% CI [0.006, 0.030], *N* = 76 (Fig. 2). The increase in *R*^2^ due to the interaction was 0.10, *F*(1,72) = 8.86, *p* = 0.004. While in AUT-P, face memory skill on the CFMT predicted their ability to understand another person’s mental states on the RMET (*b* = 0.02, SE = 0.005, *t* = 3.45, *p* = 0.001, 95% CI [0.008, 0.029], *N* = 26), this was not the case for AUT-NP (*b* = 0.0004, SE = 0.003, *t* = 0.15, *p* = 0.88, 95% CI [− 0.005, 0.006], *N* = 50).

### The relation between identity and mental state recognition does not depend on individuals’ basic face perception skills

We ran a moderated moderation model in order to investigate the role of face perception, assessed via the CFPT (see Table 4 for performance scores), on the interaction between identity recognition and prosopagnosia over participants’ ability to recognize another person’s mental state from the eye region. The CFPT was not normally distributed (significant Shapiro-Wilks test) due to a positive skew, which was resolved by square root transformation. None of the main effects nor interactions were significant. In particular, the three-way interaction between identity recognition × face perception × prosopagnosia was not significant, *b* = − 0.003, *t* (54) = − 0.41, *p* = 0.68, 95% CI [− 0.02, 0.01], *N* = 62.

**Table 4:** Group performance at the CFPT

	*N*	Error score	SD	Range
**CFPT upright AUT**	62	51.97 95% CI [45.61, 58.32]	25.02 95% CI [21.26, 30.41]	18–116
**AUT-NP**	41	44.44 95% CI [37.19, 51.69]	22.97 95% CI [18.86, 29.39]	20–96
**AUT-P**	21	66.67 95% CI [56.37, 76.96]	22.61 95% CI [17.30, 32.65]	18–116
**CFPT inverted AUT**	62	73.94 95% CI [69.97, 77.90]	15.61 95% CI [13.27, 18.98]	36–102
**AUT-NP**	41	71.61 95% CI [66.41, 76.81]	16.48 95% CI [13.53, 21.08]	36–100
**AUT-P**	21	78.48 95% CI [72.58, 84.38]	12.96 95% CI [9.82, 18.72]	50–102
**CFPT inversion effect (Inv-Up)**				
**AUT-NP**	41	27.17 95% CI [21.88, 32.47]	16.77	− 10–58
**AUT-P**	21	11.81 95% CI [2.99, 20.62]	19.37	− 32–44
**Controls from Bowles et al. 2009**	118	26.43	14.41	

*CFPT* Cambridge Face Perception Test, *AUT* autistic participants, *P* prosopagnosic, *NP* nonprosopagnosic, *SD* standard deviation, *CI* confidence interval

### Autistic individuals with prosopagnosia do not show face memory inversion effect and have no general memory difficulties

**Fig. 3:**
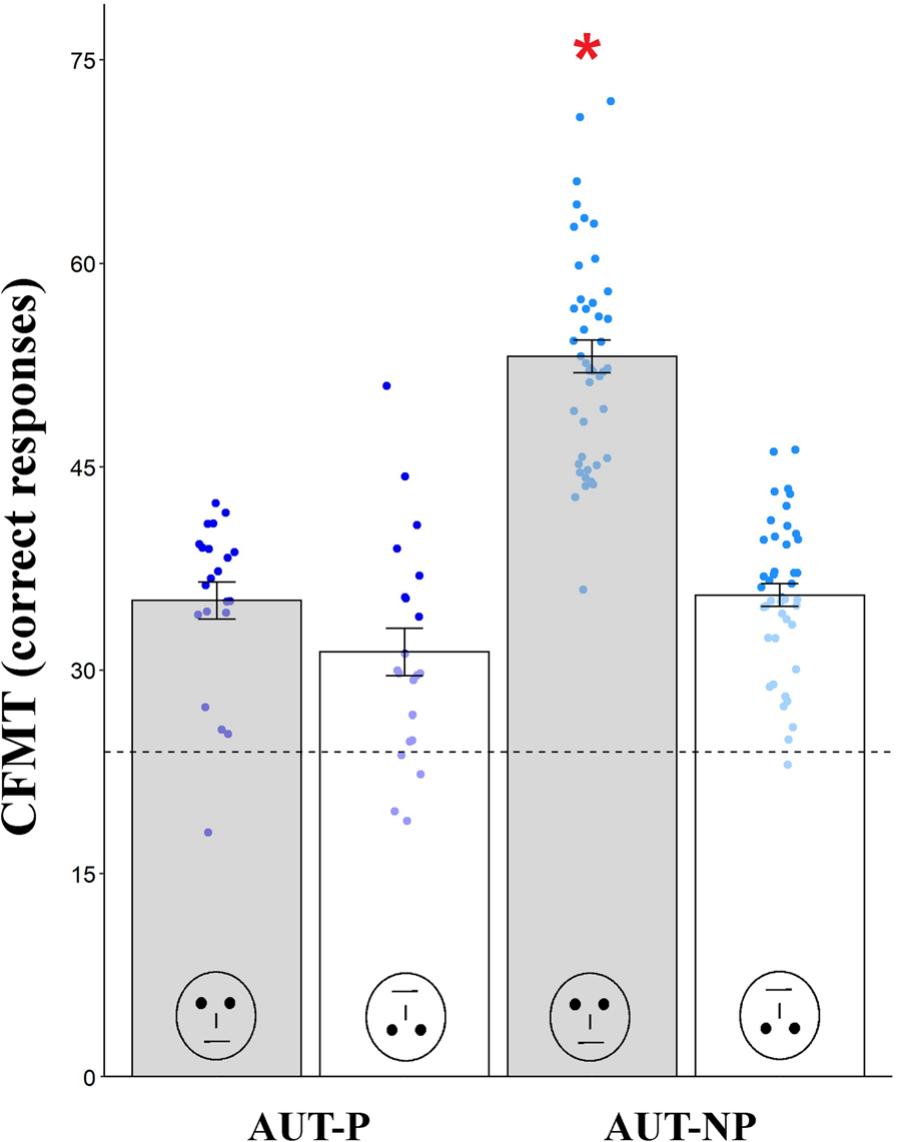
Autistic prosopagnosics do not show face inversion effect and have no general memory impairment. Non-prosopagnosic autistic participants (AUT-NP, *N* = 42, light blue dots) showed a face inversion effect, that is they performed better on upright (gray bars) vs. inverted (white bars) faces, while prosopagnosic autistic participants (AUT-P, *N* = 22, dark blue dots) did not. AUT-P did not show a general memory impairment, that is, although they performed worse than AUT-NP on upright faces, they did not differ from AUT-NP on inverted faces. The red asterisk indicates that AUT-NP performance at the upright CFMT differs from all other conditions (*p*s < 0.05). The dashed horizontal line indicates the CFMT chance level which corresponds to 24 correct responses

To further investigate differences in face processing and memory skills, we compared AUT-P and AUT-NP performance on the CFMT with upright and inverted faces (Fig. 3). Type III mixed ANOVA revealed significant main effects of prosopagnosia, *F*(1,62) = 54.30, *p* = 4.94e^-10^, *η*_*p*_^2^ = 0.47, and face orientation, *F*(1,62) = 98.82, *p* = 1.88e^-14^, *η*_*p*_^2^ = 0.61, which were explained by a significant prosopagnosia × face orientation interaction, *F*(1,62) = 41.01, *p* = 2.29e^-08^; *η*_*p*_^2^ = 0.40. Tukey HSD post hoc tests evidenced that while AUT-NP (*N* = 42) showed a face inversion effect, AUT-NP upright = 53.14 ± 7.90, AUT-NP inverted = 35.53 ± 5.55, *p* = 0.0002, AUT-P (*N* = 21) did not perform better on upright (35.14 ± 6.28) than inverted faces (31.33 ± 8.06, *p* = 0.15). AUT-P performed worse than AUT-NP on upright faces (*p* = 0.0001), but they did not differ on inverted faces (*p* = 0.11). All other post hoc comparisons were not significant (*p*s > 0.14). To test whether group performance differed from the chance level of responding, for each condition, we ran one-sample *t* tests against the chance level and found that both groups performed differently from the chance level in all conditions: upright CFMT, AUT-P: *t* (20) = 8.13, *p* = 9.04e^-8^, 95% CI [32.28, 38.00]; AUT-NP: *t*(42) = 24.20, *p* = 2.76e^-26^, 95% CI [50.71, 55.57] and inverted CFMT, AUT-P: *t*(20) = 4.17, *p* = 4.72e^-4^, 95% CI [27.67, 35.00]; AUT-NP: *t*(42) = 13.63, *p* = 5.08e^-17^, 95% CI [33.83, 37.24]. The latter result, together with the fact that only 3 AUT-P and 1 AUT-NP participants performed below the chance level on the inverted CFMT and only 1 AUT-P on the upright CFMT (Fig. 3), excluded the presence of a floor effect in performance.

AUT participants completed both the CFMT upright and inverted (in counterbalanced order) while NT participants completed only the CFMT Upright. Results of a 2 × 2 mixed ANOVA showed a non-significant main effect of order, *F*(1,62) = 0.18, *p* = 0.67, *η*_*p*_^2^ = 0.003, and a significant main effect of orientation, *F*(1,62) = 102.5, *p* = 9.21e^-15^, *η*_*p*_^2^ = 0.62, with a more accurate performance for upright compared to inverted faces. Notably, the order × orientation interaction was non-significant, *F*(1,62) = 1.62, *p* = 0.29, *η*_*p*_^2^ = 0.02, assuring that the order in which AUT participants completed the upright or inverted CFMT did not influence their performance at the upright CFMT.

### Autistic individuals with and without prosopagnosia show face perception inversion effect

To extend our investigation about differences in identity processing between AUT-P and AUT-NP to their perceptual abilities, we compared AUT-P and AUT-NP performance on the CFPT with upright and inverted faces. Type III mixed ANOVA on the CFPT error scores revealed significant main effects of prosopagnosia, *F*(1,60) = 9.75, *p* = 0.003, *η*_*p*_^2^ = 0.14, and face orientation, *F*(1,60) = 67.49, *p* = 2.11e−11, *η*_*p*_^2^ = 0.53, which were explained by a significant prosopagnosia × face orientation interaction, *F*(1,60) = 10.48, *p* = 0.002, *η*_*p*_^2^ = 0.15. Tukey HSD post hoc tests showed that both groups performed better with upright vs. inverted faces (AUT-NP: *p* = 0.0002; AUT-P: *p* = 0.02) and that only in the case of upright faces, AUT-P performed worse than AUT-NP (upright: *p* = 0.0004; inverted: *p* = 0.56). Finally, AUT-P showed a smaller perceptual face inversion effect than AUT-NP, *t*(60) = − 3.24, *p* = 0.004, 95% CI [− 24.85, − 5.87] (see Table 4).

### Face individual identity recognition is not linked to general intelligence

**Fig. 4:**
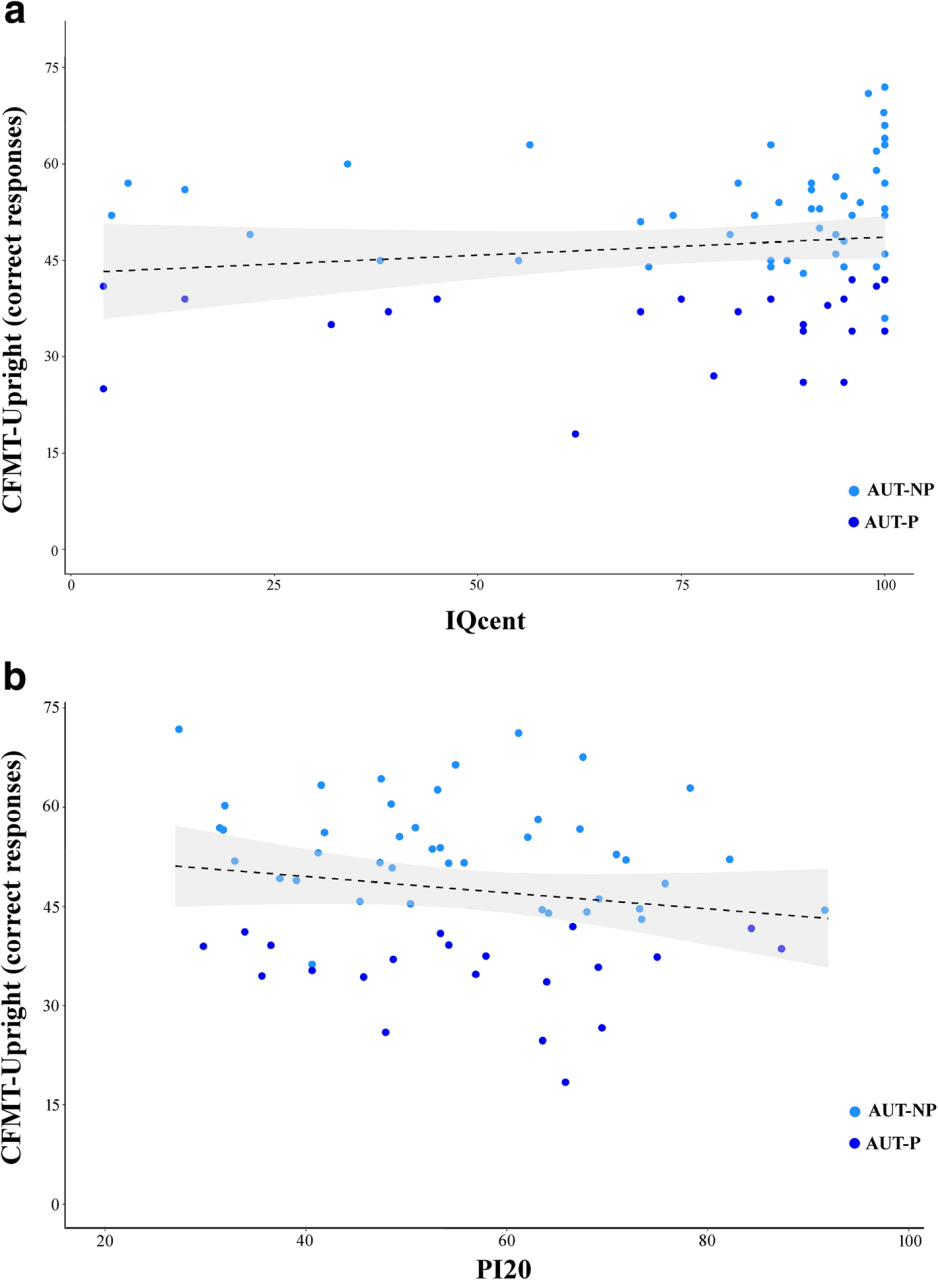
**a** General intelligence does not correlate with face memory skill in autism. In autistic participants, IQ level (percentile) does not significantly correlate with face memory skill (number of correct responses) at the Cambridge Face Memory Test (CFMT). Light blue dots represent nonprosopagnosic autistic participants (AUT-NP) while the dark blue dots represent prosopagnosic autistic participants (AUT-P). The dashed black line represents the nonsignificant regression line, while the surrounding gray-shaded area represents a 95% confidence interval. **b** Subjective face memory awareness does not predict objective face memory performance in autistic individuals. In autistic participants self-reported prosopagnosia traits at the prosopagnosia index questionnaires (PI20) do not correlate with their objective performance at the Cambridge Face Memory Test (CFMT). The light blue dots represent non-prosopagnosic autistic participants (AUT-NP) while the dark blue dots represent prosopagnosic autistic participants (AUT-P). The dashed black line represents the nonsignificant regression line, while the surrounding gray-shaded area represents a 95% confidence interval

Level of intelligence (IQ percentile) did not significantly correlate with face memory (number of correct responses on the CFMT) both in NT (Spearman *ρ* = 0.11, *p* = 0.36, 95% CI [− 0.12, 0.34], *N* = 69) and AUT (Spearman *ρ* = 0.20, *p* = 0.09, 95% CI [− 0.06, 0.42], *N* = 74) participants (Fig. 4a).

### Subjective face memory awareness does not predict objective face memory performance in autistic individuals

Regression analysis showed that AUT participants self-reported prosopagnosic traits (assessed via the PI20 questionnaire) did not predict their objective face memory/individual identity recognition skills (assessed via the CFMT), *b* = − 0.16, *t*(61) = − 1.29, *p* = 0.20, *R*^2^ = 0.03, *F*(1,61) = 1.66, *p* = 0.20, *N* = 63 (Fig. 4b).

## Discussion

Our results show that prosopagnosia potentially occurs in more than one third (i.e., 36%) of autistic adults without ID. Importantly, our prosopagnosia-based stratification into two groups, AUT-P and AUT-NP, was independent of symptom severity, autistic traits, IQ, general memory skills, empathy, and alexithymia. This is in keeping with our idea that face memory difficulties do not interact with autism at a necessary and sufficient aetiological level, an idea that is also supported by the fact that DP individuals do not have difficulties in social skills [134], nor, to the best of our knowledge, they are known to have higher rates of autism. We speculate that difficulties in face IIR may therefore not lie on the causal pathway from genes to autism. In keeping with the polygenic nature of autism [135] and probably of DP [43], difficulties in IIR may rather contribute to autism’s genetic background liability [136] in a manner resembling a multivariate correlated liability model [137] including multiple genes, endophenotypes, and environmental factors. In such a schematic model, the association between genetic risk factors of autism and those of prosopagnosia could vary from reflecting mere spatial proximity of the implicated alleles, which therefore remain linked over generations, to functional impact on the same neural circuits (e.g., the OXY system). Face IIR might be valuable to help focus research on new genetically meaningful autistic subgroups, neurobiological pathways, and neural systems. Its role may be similar to what proposed by Constantino [138] for other symptoms which are not specific to autism but which are highly prevalent in autism and are strongly genetically influenced.

We investigated the relationship between face memory and mental state recognition from the eye region as both are impaired in autism [51, 108] and rely on extracting information from the same (eye) region [139] which has strong diagnostic [87, 88] and predictive [140, 141] value to autism. Our data show that identity recognition was associated with the ability to recognize another person’s mental state by looking at their eyes, both essential skills to navigate the social world. In particular, face identity recognition was linked to mental states understanding exclusively in autistic participants who were prosopagnosic. Such association was unlikely due to reduced expertise with faces, as non-autistic individuals with DP are not impaired in mental state recognition from the eye region [103, 114–116]. Alternatively, reduced attention to the eyes may affect both identity [139, 142–144] and mental state recognition and may be linked to altered OXY-mediated social processing of identity sensory cues [31, 45]. The chances of a common neurobiological mechanism underlying both face identity and mental state recognition in AUT-P increase the potential relevance of IIR as an endophenotype, as it stratifies autistic individuals in a way that is meaningful to distinctive difficulties in social interaction. The differential relationship we found in AUT-P and AUT-NP between face IIR and mental state recognition from the eye region supports the idea that AUT-P and AUT-NP might be two separate subgroups, and not just that AUT-NP were autistic individuals with poor identity recognition skills [125]. We included performance on the Cambridge Face Perception Test as an additional moderator to investigate whether difficulties in face identity and emotion recognition in AUT-P were both due to perceptual failure in face processing. We found that the relationship between identity and mental state recognition was not moderated by altered basic face perception skills that impair face recognition even when face memorization is not needed (i.e., apperceptive prosopagnosia).

To further investigate between-group differences in face processing and general memory skills, we tested autistic participants’ memory for inverted faces, which, unlike from upright faces, are typically not processed holistically [145]. In their review, Weigelt and colleagues [63] reported that only 2 out of 14 case-control studies showed no face inversion effect in autistic participants, and so, the majority of studies suggest better face recognition for upright vs. inverted faces [102] also in autistic individuals. However, none of these studies controlled for prosopagnosia. Our results show that while AUT-NP remembered faces better when presented upright vs. inverted, AUT-P did not show such face inversion effect. This suggests that upright faces may not be a special class of stimuli to remember for AUT-P and that presence of prosopagnosia, not autism, drove the lack of face memory inversion effect as both groups had an autism diagnosis. Interestingly instead, both AUT-NP and AUT-P showed face inversion effect when their face perception skills were assessed with no memory demands (i.e., on the CFPT), again suggesting that retention of facial identity information is crucial in differentiating AUT-P from AUT-NP. Nonetheless, interpretation is not so straight forward as results on the presence of face inversion effects in DP are mixed, evidencing holistic face processing is not always impaired in DP [43, 101]. The absence of face memory inversion effect and a positive correlation between face memory and mental state understanding are instances in which AUT-P differed from DP. It remains an open question whether prosopagnosia in AUT shares similar characteristics and neurobiological correlates with prosopagnosia in non-autistic, DP individuals. In addition, we found that, while AUT-P performed worse than AUT-NP with upright faces (at both perceptual and memory levels), they did not differ with inverted faces, suggesting our results were not due to unspecific memory impairments.

As IQ varies greatly along the autistic spectrum [146], we checked whether IQ correlated with IIR and found that this was not the case both for our AUT and NT participants, a result similar to that found in the general population [78, 79]. Face memory may therefore be potentially relevant to the entire autistic spectrum, across all levels of intellectual ability. It remains to be tested whether the independence between face memory skills and IQ holds true also with individuals with ID.

Lastly, we explored autistic participants’ awareness of their face IIR skills and whether a self-report questionnaire, here the PI20 questionnaire, could be used for screening autistic individuals with difficulties in face memory. Contrary to the general population and to DP individuals [120–122], subjective scores of autistic participants on the PI20 did not predict their objective performance on the CFMT. Therefore, a self-report questionnaire does not seem a reliable prosopagnosia screening tool for autism, which is at odds with previous findings showing that adult autistic participants had similar face memory awareness compared to neurotypical individuals [147].

### Limitations

This was not an epidemiological sample and therefore may not be representative of the prevalence of prosopagnosia in autism. However, it is the largest sample available to date and it was not biased with respect to face processing skills, so it may indeed reflect the true prevalence of prosopagnosia in autistic adults with no ID. We do not know whether our results generalize to the entire autism spectrum. Future work is needed to determine whether prosopagnosia is equally prevalent and similarly associated with mental state recognition skills also in other autistic individuals not represented in the current sample, such as in children, individuals with ID and females (our sample included only 20% of females).

Our proposal that prosopagnosia might be a potential endophenotype in autism would benefit from additional findings supporting co-segregation of autism and prosopagnosia within families and higher rate of prosopagnosia in non-autistic family members compared to the general population.

### Future perspectives

Autism research benefits from parallel human and animal studies and our results, taken in the context of the current literature, open various avenues of research. Future human studies could investigate whether face memory difficulties can subgroup autism high-risk infants in prospective meaningful ways. Given that intranasal OXY (INOXY) was shown to normalize identity recognition in DP [44], correctly increase familiar judgments of previously seen faces in controls [148], and improve eye contact in autistic individuals [149], researchers could investigate its effects on face memory in autism. Researchers could also investigate, as a downstream cascade, the effects of INOXY on other face memory-related social behaviors relevant to autism, such as social anxiety and attentional preference for faces [150, 151]. Further, since face memory could be a proxy to autistic participants’ OXY-relevant genetic background, researchers could investigate whether it predicts responders to INOXY. This face IIR-based stratification of participants may help addressing the failure of many [152, 153], often underpowered [154], INOXY intervention studies with autistic participants.

Recent evidence showing that assessment of IIR in autistic individuals may extend beyond visual-face to other sensory systems and identity-conveying cues such as auditory-voice [155] and olfactory-body odor [156], increases the possibility to directly translate experimental paradigms and research questions to autism rodent models [85]. Animal studies, overcoming limitations intrinsic to human research, could examine if and which genetic autism mouse models show IIR deficits, to then uncover their neuro-biological correlates with a focus on OXY’s possible modulatory role in generating states for optimized information extraction [157] and in attributing salience and reward to identity-relevant sensory cues [158].

In conclusion, we found that difficulties in face individual identity recognition are highly prevalent in autism and are linked to difficulties in mental state understanding from the eye region independently from face perception skills. Further, they stratify autistic individuals irrespective of intellectual ability, diagnostic symptoms, and personality traits. Because of its potential role as an endophenotype, we believe individual identity recognition may be important to advance our understanding of autism within a translational framework informed by, and informative to, neurobiological non-human animal research.

## Data Availability

The anonymized datasets analyzed in the current study are available from the corresponding author on request.

## Abbreviations

ADI-R: Autism Diagnostic Interview Revised
ADOS: Autism Diagnostic Observation Schedule
AQ: Autism spectrum quotient
AUT: Autism
AUT-NP: Autistic non-prosopagnosic
AUT-P: Autistic prosopagnosic
CFMT: Cambridge Face Memory Test
CFPT: Cambridge Face Perception Test
CI: Confidence interval
CSS: Calibrated Severity Score
EC: Empathic concern
ID: Intellectual disability
IIR: Individual identity recognition
INOXY: Intranasal oxytocin
IQ: Intelligence quotient
IRI: Interpersonal Reactivity Index
NT: Neurotypical
OXY: Oxytocin
PI20: Twenty-item Prosopagnosia Index
PT: Perspective taking
RRB: Restricted Repetitive Behaviors
SA: Social affective
SD: Standard deviation
SE: Standard error
TAS: Twenty-item Toronto Alexithymia Scale
VIF: Variance inflation factor
